# Enhancing the best-first-search F with incremental search and restarts for large-scale single machine scheduling with release dates and deadlines

**DOI:** 10.1007/s10479-024-06386-7

**Published:** 2024-11-21

**Authors:** Yacine Laalaoui, Rym M’Hallah

**Affiliations:** 1 Information Technology Department, Taif University, Taif, Kingdom of Saudi Arabia; 2 Engineering Department, King’s College London, S2.41 Strand Building, Strand, London, WC2R 2ND UK

**Keywords:** Scheduling, F Heuristic search, Release dates, Deadlines, Restarts, Incremental Search

## Abstract

This paper presents a new heuristic search $\hbox {F}_{IR}$ that determines the feasibility of scheduling a set of jobs on a single machine where each job is characterized by its release date, processing time, and deadline. $\hbox {F}_{IR}$ uses incremental search and restart strategies to scale the best-first-search heuristic F (Laalaoui and M’Hallah, in: IEEE symposium on evolutionary scheduling and combinatorial optimisation (SSCI) 2022, IEEE, Singapore, 2022). Experimental results provide computational evidence of the superiority of $\hbox {F}_{IR}$ for large-scale instances. In fact, $\hbox {F}_{IR}$ outperforms the IBM ILOG CP constraint programming solver, the CPLEX mixed integer programming solver, and existing heuristics. It finds feasible solutions for many instances with 100,000 jobs in less than one minute.

## Introduction

The new global economic order with its more efficient logistics and flexible supply chains has altered production from off-line demand, with long lead time, high-volume, and a limited assortment of products to responsive, low volume, high-mix make-to-order production. In this emerging environment, manufacturers must accept or reject orders almost instantly. Undertaking such decisions relies on identifying a feasible solution to large scale scheduling problems where orders are characterized by release times and deadlines.

### Problem statement

This paper addresses the simplest core decision problem ${\mathfrak {P}}$ that arises in order acceptance (Nguyen et al., 2015). Consider a set $\Pi$ of *n* jobs, where job $j, j\in \Pi ,$ is characterized by its positive integer release date $r_j,$ processing time $p_j,$ and deadline $d_j.$ ${\mathfrak {P}}$ determines whether there exists a feasible schedule of the *n* jobs without preemption on a single machine that processes one job at a time. In a feasible schedule, every job *j* starts after its release time and after the completion time of its immediate predecessor, and completes before its deadline. When such a sequence exists, every job $j,\ j \in \Pi ,$ is “on-time”  and none is “tardy”. As such, ${\mathfrak {P}}$ is equivalent to mapping each job $j \in \Pi$ into a unique position $k\in K$ where *j* is on-time.

In Graham’s three-field notation for scheduling problems (Graham et al., 1979), ${\mathfrak {P}}$ is denoted as $1|r_j|L_{max}$ where $L_{max}\le 0$. The first field denotes the processing environment, which is a single machine. The second field indicates the job characteristics, but only deviations from standard assumptions are signalled. In this case, each job has a release time $r_j$. The third field is the measure of performance, which is the maximum lateness $L_{max}$. When optimizing the scheduling problem yields $L_{max} \le 0,$ the decision problem ${\mathfrak {P}}$ is feasible. The scheduling optimization problem $1|r_j|L_{max}$ is known to be ${\mathcal {N}}{\mathcal {P}}$-Hard (Lawler et al., 1993). Alternatively, ${\mathfrak {P}}$ is denoted as $1|r_j|\sum U_j$ where the number of tardy jobs given by $\sum U_j=0$ and $U_j=1$ if job *j* is tardy and 0 otherwise. “No approximation algorithms with a provable approximation factor are known”(Maack et al., 2024) for $1|r_j|\sum U_j$, which is ${\mathcal {N}}{\mathcal {P}}$-hard (Lawler et al., 1993).

### Relevance

${\mathfrak {P}}$ is relevant both in academia and industry. In academia, ${\mathfrak {P}}$ is a challenging Artificial Intelligence and Operations Research problem. Its ${\mathcal {N}}{\mathcal {P}}$-hard nature makes solving its difficult large instances a real challenge. Yet, ${\mathfrak {P}}$ is a subproblem of both classical parallel machine (M’Hallah & Al-Khamis, 2012; Polyakovskiy & M’Hallah, 2014), flow-shop (M’Hallah, 2014; M’Hallah & Bulfin, 2007), and job-shop problems. In addition, it is a component of non-traditional complex scheduling problems with deadlines such as the decentralized heterogeneous machine scheduling problem (Lang et al., 2016), and a relaxation to more complex problems such as batch manufacturing (Nascimento et al., 2024; Polyakovskiy & M’Hallah, 2021; Rocholl & Mönch, 2024), delivery (Letsios et al., 2021), and unloading operations in train terminals (Kouismi et al., 2016). Furthermore, ${\mathfrak {P}}$ appears whenever a machine must be rescheduled because of delayed release times of jobs or tighter deadlines imposed by (i) changes of deliveries and logistics as in rescheduling of dynamically arriving jobs that require off-site and on-site construction coordination (Clautiaux et al., 2023; Liu et al., 2024) or (ii) rescheduling of urgent / priority jobs (Xiong et al., 2024), or (iii) integration issues in the supply chain (Aminzadegan et al., 2019), or (iv) rolling time horizon approaches (Detienne, 2014).

In industry, ${\mathfrak {P}}$ is core to order acceptance (Nguyen et al., 2015). For instance, manufacturing and service workshop managers assess the feasibility of honouring their contractual on-time deliveries and plan their production and maintenance using variants of ${\mathfrak {P}}$. For example, in a car-repair shop, a technician can be assimilated to a single processor and requests for car repair are either accepted or deferred. In an emergency surgical department, each operating room can be assimilated to a single machine and the decisions whether to accept or divert a patient are undertaken. In ore extraction (Agnetis et al., 2006), the strategic management of the supply chain involves large scale scheduling problems whose optimization resorts to solving ${\mathfrak {P}}$ at each of the five stages of the supply chain: block mining, transportation of mined raw material to factory, ore manufacturing, holding in warehouse, and delivery to client. These five sequential tasks are constrained by respective release dates and deadlines. In cloud computing (Mahmood et al., 2017), parallel computers receive processing requests from billions of jobs. Each job is characterized by its release time and its deadline. Even though such a deadline may not be expressed explicitly, it is usually estimated based on historical data or observed clients’ behaviour. It reflects the clients’ perceived waiting time beyond the usual processing time. In the banking sector, very large volumes of transaction requests must be processed within a very tight time window, set by central banks or ISO standards (SWIFT). Last, real-time cyber-physical systems (Park et al., 2014; Shen et al., 2016) are communication-enabled autonomous systems that are used in smart grids, driver-less autonomous vehicles, train transportation, medical monitoring, process control systems, and automatic pilots. These systems use a large amount of distributed computing power to manage the information they receive from their multiple sensors and actuators. How to manage their computing power to process all received information in a timely manner is a difficult task. This requires scheduling large-scale real-time task-sets under efficiency and efficacy constraints.

In summary, ${\mathfrak {P}}$ is a timely and highly relevant industrial problem. In addition to its academic attractiveness, it appears as a sub-problem of more complex scheduling problems or as a relaxation/simplification of many real life problems.

### Scientific challenges

Small easy instances of ${\mathfrak {P}}$ can be modelled as a mixed integer program (MIP) and solved via IBM CPLEX or Gurobi. MIP solvers apply a special purpose Branch-and-Bound (B&B) (Xu & Parnas, 1990). Similarly, ${\mathfrak {P}}$ can be modelled as a constraint satisfaction program (CSP) and solved via a CSP solver such as the IBM ILOG CP, which adopts a maintaining arc consistency (MAC) strategy. MAC is based on a depth first search (DFS) augmented with constraint propagation techniques. However, neither MIP nor CSP solvers are viable approaches for large-sized difficult instances; in particular, for those with highly clustered narrow time windows. This is expected: all exact approaches for this ${\mathcal {N}}{\mathcal {P}}$-hard problem have an exponential time complexity while being limited by storage availability. For instance, the storage space complexity of MIP is exponential while that of MAC-based approaches changes from polynomial to pseudo-polynomial when the CSP solver incorporates filtering algorithms (van Dongen, 2002).

In summary, existing exact approaches do not scale well to large-scale instances of ${\mathfrak {P}}$ such as those occurring in urban systems (Chen et al., 2017), production systems (Baumann & Trautmann, 2014), mega-projects’ management (Krishnamoorthy et al., 2012), and cyber-physical systems (Park et al., 2014; Shen et al., 2016). In fact, their performance is unfit in these circumstances not only in terms of solution quality but most importantly in terms of run time. Hence, as real-life problems where ${\mathfrak {P}}$ arises are getting larger and more complex, solution approaches to ${\mathfrak {P}}$ must become more efficient.

### Contribution

This paper proposes a fast approach, labelled $\hbox {F}_{IR}$, for large-scale instances of ${\mathfrak {P}}$. $\hbox {F}_{IR}$ enhances the search heuristic F (Laalaoui & M’Hallah, 2022) with multiple-restarts and incremental-search. F, which is an iterative two-phase approach, alternates between constructing a partial feasible solution and backtracking. During its construction phase, F assigns positions to on-time jobs, with jobs ordered according to their merit values. When it can’t schedule the current job on-time, F initiates its backtracking phase, which removes the on-time jobs that are causing the tardiness of the current job. F stops when it either successfully schedules all jobs or reaches a threshold run time.

$\hbox {F}_{IR}$ enhances F in two ways. First, it augments F with an incremental search that allows the gradual resolution of the problem. This search applies a divide-and-conquer strategy with an iterative call to F to solve *n* subproblems. Each call appends one additional job to the current schedule (obtained by prior calls). The search stops when a feasible sequence of the *n* jobs is found or a threshold run time is reached. Second, $\hbox {F}_{IR}$ augments F with a restart mechanism that explores the state-space and finds feasible solutions by aborting the search in non-leading directions after a threshold time. When it explores a search direction but fails to find a feasible solution within the allocated time, F concludes that it is most likely searching in a wrong direction. Thus, F aborts its search in this direction and restarts in a different random direction. While the divide-and-conquer strategy enhances the efficiency of F, the restart strategy offers a better exploration of the state-space by better managing run time. These strategies allow $\hbox {F}_{IR}$ to solve instances with more than seven thousand jobs in a few seconds. The IBM CP solver takes more than 18 h to identify a feasible solution for these instances, which are intractable by CPLEX and Gurobi alike.

### Outline

Section 2 reviews the literature for the decision problem ${\mathfrak {P}}$ and for related single machine scheduling problems with deadlines. Section 3 presents F and illustrates its application on an example. Sections 4 and 5 detail the two enhancement mechanisms of F. Section 6 analyzes the outcomes of the experimental investigation undertaken on newly proposed instances that will serve as a benchmark set. Finally, Sect. 7 summarizes the paper and enumerates potential research directions.

## Literature review

The feasibility of ${\mathfrak {P}}$ can be determined by solving $1|r_j|\sum U_j$ and checking whether $\sum U_j=0.$ Special-purpose exact algorithms for $1|r_j|\sum U_j$ are based on Branch-and-Bound; thus, are not scalable beyond a couple of hundred jobs because of memory limitations. This includes three best known exact algorithms: (i) the best exact Branch-and-Bound of M’Hallah and Bulfin (2007) for the weighted case where bounds are obtained through a surrogate relaxation that results in a multiple-choice knapsack, (ii) the Branch-and-Bound-and-Remember of Kao et al. (2012) for the unweighted case, where cyclic best first search explores dominance properties and bounding techniques to reduce the search space, and (iii) the MIP based model with additional valid inequalities, bound tightening constraints, and lifted knapsack cover cuts of Detienne (2014) and which solves randomly generated instances with up to 500 jobs in one hour of runtime using the off-the-shelf solver IBM CPLEX.

${\mathfrak {P}}$ can be modelled as a MIP. MIP solvers use Branch-and-Bound techniques with exponential space complexity; thus, are limited in terms of problem size when solving ${\mathfrak {P}}$. Generally (to the best of the authors’ knowledge), MIP-based solvers are geared toward optimization problems rather than CSPs. For example, the satisfiability (SAT) problem, which is a representative CSP, is not addressed via MIP but using backtracking procedures. When modelled as a CSP, ${\mathfrak {P}}$ can be solved using off-the-shelf IBM ILOG CP solver. A possible CSP model uses the jobs as variables and unary constraints to represent the time windows (Baptiste et al., 2001). Finding a feasible solution to this *disjunctive scheduling* problem ${\mathfrak {P}}$ is equivalent to identifying feasible values for a set of *n* variables. Each job *j* corresponds to a variable whose domain of possible values depends on $r_j,\ p_j$, and $d_j$. A solution is feasible to ${\mathfrak {P}}$ if it satisfies all timing constraints.

${\mathfrak {P}}$ was solved approximately using (meta) heuristics such as NEH heuristic, Profile Fitting (PF) (McCormick et al., 1989), squeaky wheel optimization (SWO) (Joslin & Clements, 1999), ant colony optimization (ACO) (Laalaoui et al., 2009), learning in hard real-time scheduling with double learning (LHRTS-DL) (Laalaoui & Drias, 2011), and F (Laalaoui & M’Hallah, 2022). NEH was proposed for the minimal makespan *m*-machine flowshop scheduling problem, but has been extended to other performance criteria and other manufacturing environments. It considers the *n* jobs in a non-increasing order of a given criterion. For a flow shop, this criterion is the total processing time of a job. NEH schedules the first job, and sets $k=1$. It inserts job $k+1$ into every position $k',\ k'=1,\ldots ,k+1$; thus right-shifting all jobs positioned in subsequence $k',\ldots ,k+1$. For job $k+1$, NEH chooses the position that yields the least objective function value of the $k+1$ positioned jobs. It fixes the partial sequence of the first $k+1$ jobs and increments *k*. It stops when all jobs are positioned. NEH considers $\frac{n(n-1)}{2}-1$ (sub)sequences of which only *n* are sequences of the *n* jobs. Only the sub-sequence of the two first positioned jobs is guaranteed to be optimal. That is, NEH is an insertion heuristic that performs an almost blind search with no guarantee of local or global optimality. Even though the selection/ordering criterion seems natural for the flow shop problem, it is not as clear for other criteria, in particular for due date related ones. Despite its $O(n^2)$ complexity, NEH is computationally expensive. Its high overhead cost is caused by the right shifting of the jobs at every insertion. Thus, it is relatively slow.

PF was designed to minimize the cycle time of a flow shop assembly line with no buffer capacity between the machines. This problem is equivalent to minimizing the makespan of the no-wait flow shop. PF positions the job with the longest processing time in the first position of the sequence and sets $k=1$. Among the remaining $n-k$ jobs, PF assigns the next position of the sequence to the job that yields the smallest non-productive time (idle and blocking time) in the partial schedule of the $k+1$ positioned jobs. PF’s selection of the jobs is adapted according to the objective function of the problem.

SWO is a general optimization framework that consists of three cyclic steps. It uses a priority rule to build a complete solution. Next, it analyzes the obtained solution: It assesses the solution value and identifies areas of conflict or of potential improvement of its objective value. Finally, it updates the priority list taking into consideration the information extracted from the second step. It then restarts a new cycle using the updated information. It stops when it proves optimality or reaches a preset number of cycles. This framework is tested on parallel machine scheduling with release times and deadlines and on graph coloring problems. The former reduces to ${\mathfrak {P}}$ when the number of machines is one. A major drawback of SWO is that it handles $n-$job sequences. It builds a permutation of *n* jobs, identifies the tardy ones, and uses this information to find a different permutation. However, repairing one conflict in an ad-hoc manner may, in turn, engender new conflicts.

Unlike the complete-and-repair SWO, ACO and LHRTS-LH are constructive. They manipulate partial solutions that consist of on-time jobs. Consequently, a conflict may occur only when a non-scheduled (or free) job *j* is appended to the current solution $\mathcal {S}$. In such a case, one or more jobs are removed from $\mathcal {S}$ in hope of inserting *j* into a non-conflicting position during the next iteration. In ACO, each ant adds iteratively a free job to its path. It stops appending jobs if the last annexed one is tardy or all jobs are sequenced. At each iteration, it selects the job to append either deterministically or stochastically. It bases the deterministic selection on a weighted sum of the pheromone levels, a merit value, and a distance function. The merit function *f*(*j*) of a job *j* determines when *j* is to be appended to the path of the ant. The distance function $h_2(j)$ of *j* reflects the number of ants that have chosen to append *j* but refrained from annexing *j* because *j* can’t be on-time. Thus, the larger the number of ants facing the infeasibility of scheduling *j* on-time, the higher $h_2(j)$ is, and the higher the chances are that *j* is chosen sooner by other ants (Laalaoui et al., 2009). The stochastic selection ponders the weight of the information received from the pheromone levels and merit values. However, this selection mechanism doesn’t prevent ants from undertaking myopic decisions while the pheromone levels can’t effectively and efficiently guide the ants’ movements.

LHRTS-DL selects each job *j* using a merit function $f(j)=h_1(j) -h_2(j)$. At each iteration, it rebuilds $\mathcal {S}$ from scratch; thus either obtains a feasible solution or refines its knowledge about the merit values of the jobs; an information that it uses to eventually converge to a feasible schedule of the *n* jobs. However, rebuilding $\mathcal {S}$ from scratch, at each iteration, slows LHRTS-DL. In addition, $h_2(j)$ may be infinite because $h_2(j)$ is increased every time *j* is selected and turns out to be tardy. In such a case, there is no bound on the number of times *j* is selected. Yet, it is not realistic to correct a job’s position by an infinite value. To move to the first position, a job *j* in position $k,\ j \in \Pi =\{1,\ldots ,n\},\ k \in \Pi ,$ should have $h_2(j) \le k \le n.$ That is, $h_2(j)$ must be finite.

Search heuristic F, detailed in Sect. 3, improves LHRTS-DL while offering six features that distinguish it from LHRTS-DL and from existing iterative constructive backtracking techniques.

1. **The selection rule** of F chooses jobs according to a merit value that is based on the job’s current position and the job’s corrective value. Both the position and the corrective values are independent from the instance being solved. Existing approaches, on the other hand, use variable ordering, and problem dependent heuristics to select jobs.
2. **The construction phase** of F stops when either F can’t position the current job on-time or the current partial solution violates a theoretical property on the order of scheduled on-time jobs according to their merit values. This second stopping condition is not applied by existing heuristics while exact approaches, such as CSP solvers, stop when they reduce the domain of a free variable to the empty set.
3. **The backtracking phase** of F destroys the last part of the current partial schedule while preserving the first part for future use. Thus, F avoids rebuilding solutions from scratch. This feature significantly reduces the search time of F and distinguishes F from existing search algorithm, which remove all the jobs of the current partial schedule (Laalaoui & Drias, 2011). The backtracking of F uses the merit function to choose, from the partial solution, the job to be removed. Unlike existing backtracking methods, F doesn’t follow a systematic removal of the jobs. Exact approaches (Chen & van Beek, 2001; Dechter & Frost, 2002; Beek, 2006; Lecoutre et al., 2009) avoid chronological backtracking by using some kind of back jumping. For example, the backtracking of Patterson et al. (1990) is a DFS-based B&B implicit enumeration that may guarantee optimality. It is similar to the backtracking scheme of the IBM ILOG CP solver except that the solver augments its search engine with some propagation techniques.
4. **The Random exploration of the state-space** of F avoids the systematic scanning of the search tree that is applied in exact approaches and that makes them time-limited.
5. **The finite values of the merit function** allow F to solve large scale instances. They make the corrective actions of F more realistic and meaningful than those applied by other heuristics (Heilmann, 2001; Joslin & Clements, 1999).
6. **The interaction** of the above five features of F to select jobs, stop the construction phase, and initiate the backtracking phase is ensured via the jobs’ merit values. To the authors’ best knowledge, the dynamic update of finite merit values exists in no other state-of-the-art approach.

In addition, F avoids three heuristic’s drawbacks.

1. Unlike existing approaches, which are probabilistic, F uses a deterministic technique to select the next job. The unique stochastic behavior in F remains in breaking tie situations only.
2. Unlike meta-heuristics, which require tuning and whose performance depends on the tuned parameters, F is parameter-free. Thus, F avoids the parameters’ tuning phase required by meta-heuristics.
3. While existing meta-heuristics guide the search via problem-dependent heuristics, the search of F is totally independent from the instance under consideration; a property that defines its uniqueness in the class of heuristics. In computational intelligence, all (meta-)heuristics except DFS and breadth-first search use an informed search based on the input of the instance.

The proposed $\hbox {F}_{IR}$ is an enhanced version of F. $\hbox {F}_{IR}$ builds on the strengths of F while it mitigates its long run times when the number of jobs is very large. This is elucidated in Sects. 4 and 5.

## Search Heuristic F

F is an iterative two-phase approach. The constructive phase schedules the jobs according to a dynamic priority rule that reflects the merit *f*(*j*) of positioning a job *j*. The backtracking phase destroys the current partial solution for possible repair by the constructive phase. Sections 3.1 and 3.2 detail the computation of *f* and detail the backtracking mechanism. Sections 3.3 details the steps of F.

### Selection of jobs

Let $\mathcal {S}$ denote the current partial feasible sequence of on-time jobs, and $\mathcal {O}=\Pi \setminus \mathcal {S}$ the set of free jobs. F selects jobs from $\mathcal {O}$ in increasing order of their *f* values with ties broken randomly. The merit value *f*(*j*) is the difference between the position value $h_1(j)$ and the corrective action value $h_2(j)$ of a job $j,\ j \in \Pi .$ That is, $f(j)=h_1(j)-h_2(j).$

**Position Value**

$h_1(j)$ is the outcome of an integer function $h_1$ that maps each job $j,\ j \in \Pi ,$ into a unique position $k=h_1(j) \in K.$

$$\begin{aligned} h_1: \Pi \mapsto K \\ j \mapsto k. \end{aligned}$$

F assigns a position $h_1(j)$ for every job $j,\ j \in \Pi .$ F keeps $h_1(j)$ unchanged if *j* is tardy, but alters $h_1(j)$ if *j* is on-time in a new position. Because each position is assigned a single job and each job is tagged to a single position, $h_1$ satisfies the following property.

#### Property 1

$\forall$ $(i,j) \in \mathcal {S}^2$ such that *i* has been selected before $j,\ h_1(i) < h_1(j).$

Based on Property 1, the last job *j* that F appends to $\mathcal {S}$ has $h_1(j)=n.$ That is, F obtains a feasible schedule to ${\mathfrak {P}}$ when a job *j* has $h_1(j)=n.$

**Corrective Action Value**F constructs $\mathcal {S}$ by iteratively appending jobs. When F can’t schedule a job *j* on-time in a position $k,\ k \in K,$ it moves *j* backward to a position $k',\ k'=1,\ldots ,k-1;$ i.e., it corrects the position of *j* by $k-k'$. Thus, $h_2$ is an integer function that maps a job *j* into a corrective value $h_2(j) \in K'=\{0,\ldots ,n-1\}$.

$$\begin{aligned} h_2: \Pi \mapsto K' \\ j \mapsto k'. \end{aligned}$$

Initially, F assumes that all jobs can be on-time; thus, sets $h_2(j)=0$ for all $j,\ j \in \mathcal {O}.$ During its iterative steps, F sets $h_2(j)=0$ for any job *j* that can start on its $r_j$ because it will necessarily be on-time; thus, *j* doesn’t need to be moved to prior positions. For illustration purposes, consider Example 1.

**Example 1**. Consider 6 jobs where $\mathcal {S}=\{1,\ldots ,5\}$ and $\mathcal {O}=\{6\}.$ Initially, $h_2(6)=0.$ When it assigns job 6 on-time to position 6, F keeps $h_2(6)=0$. On the other hand, when this assignment makes job 6 tardy, F sets $h_2(6)=5,\ \mathcal {S}=\{1,\ldots ,4\}, \mathcal {O}=\{6,5\},$ and tries assigning job 6 on-time to position 5. If it fails, F further decreases $h_2(6),$ removes the last job of $\mathcal {S},$ appends the removed job to $\mathcal {O},$ and repeats its assignment test. F stops this iterative process when it succeeds in positioning job 6 on-time. $\square$

**Merit Value**

The merit function *f* is a mapping

$$\begin{aligned} f: \Pi \mapsto K' \ \ \ \ \ \\ j \mapsto h_1 - h_2. \end{aligned}$$

It satisfies the following property.

#### Property 2

$\forall \ (i,j) \in S^2$ such that *i* has been selected before $j:\ f(i) \le f(j).$

### Backtracking

During its constructive phase, F may encounter a job $j,\ j \in \mathcal {O},$ such that *j* is on-time when appended to $\mathcal {S}$ but *j* violates Property 2; i.e., $f(j) < f(j')$ where $j'$ is the last job in $\mathcal {S}$. In such a case, F stops its current iteration (without appending *j* to $\mathcal {S}$) and backtracks: It removes some jobs from $\mathcal {S}$ and inserts them back into $\mathcal {O}$. The backtracking scheme may be either weak or strong.

**Weak Backtracking Scheme (WBS)**

F removes $j'$ from $\mathcal {S}$, inserts $j'$ into $\mathcal {O}$, and tests the validity of Property 2. When either $\mathcal {S}=\emptyset$ or Property 2 is satisfied, F stops. Otherwise, F restarts the construction phase with the modified $\mathcal {S}$ and $\mathcal {O}$.

Consider again Example 1 with $\mathcal {S}=\{1,\ldots ,5\},\ \mathcal {O}=\{6\},\ f(1)=1,\ f(2)=f(3)=2,\ f(4)=3,\ f(5)=4$ and $f(6)=2$. F can’t append job 6 to $\mathcal {S}$ because $f(6)<f(5).$ It applies WBS, which removes iteratively jobs 5 and 4 from $\mathcal {S}.$ F then restarts the constructive phase with $\mathcal {S}=\{1,2,3\}.$

**Strong backtracking scheme (SBS)**

F resorts to WBS once it applies SBS and encounters a job $j'$ that can’t be further moved backward; i.e., $h_2(j')=0$ because $s_{j'}=r_{j'}$. In such a case, F positions *j* into the first feasible position that succeeds $j'$. When

$$\begin{aligned} (f(j') \le f(j)\ and\ h_2(j')=0)\ \ or \ \ ( \mathcal {S}=\emptyset ), \end{aligned}$$

F stops SBS and offers each job removed from $\mathcal {S}$ the chance to eventually be scheduled on-time. The removed jobs have $h_2 > 0$. Their selection in future iterations will make them tardy; i.e., will increase their $h_2$ corrective values and decrease their *f* values. This improves their chance to start on their release dates. That is, a job *j* that violates SBS needs to be rescheduled closer to its $r_j$, which is its earliest starting time.

For illustration purposes, consider Example 1 with $\mathcal {S}=\{1,\ldots ,5\},\ \mathcal {O}=\{6\},\ f(1)=1,\ f(2)=2,\ f(3)=f(4)=3,\ f(5)=5,\ f(6)=3$ and $h_2(1)=h_2(2)=h_2(3)=0,\ h_2(4)=1,$ and $h_2(5)=0$. F can’t append job 6 to $\mathcal {S}$ because $f(6)<f(5).$ The backtracking phase removes job 5 from $\mathcal {S}$ because $f(6)<f(5)$. Even though $f(4)=f(6),$ F removes job 4 from $\mathcal {S}$ because $h_2(4)=1 \ne 0.$ Then, F stops SBS because $f(3) = f(6)$ but $h_2(3) =0$; i.e., Property 2 is satisfied. Consequently, F initiates the next iteration with $\mathcal {S}=\{1,2,3\}.$

### Algorithm description

Algorithm 1 gives the pseudo code of F. Its input is the set $\Pi$ of jobs to be scheduled, the position and correction values $(h_1, h_2)$, and the allocated run time $time\_out$. Its output is a schedule $\mathcal {S}$. When F is a standalone heuristic, $\mathcal {O}=\Pi ,\ \mathcal {S}=\emptyset ,$ and both $h_1$ and $h_2$ are local variables initialized internally to $h_1=1$ (because all jobs are candidates for position 1) and $h_2=0$ (because no job is yet tardy). On the other hand, when called from an external procedure, as in Sects. 4 and 5, F is fed $Q,\ \mathcal {S},$ and values of $h_1$ and $h_2.$

**Algorithm 1:**
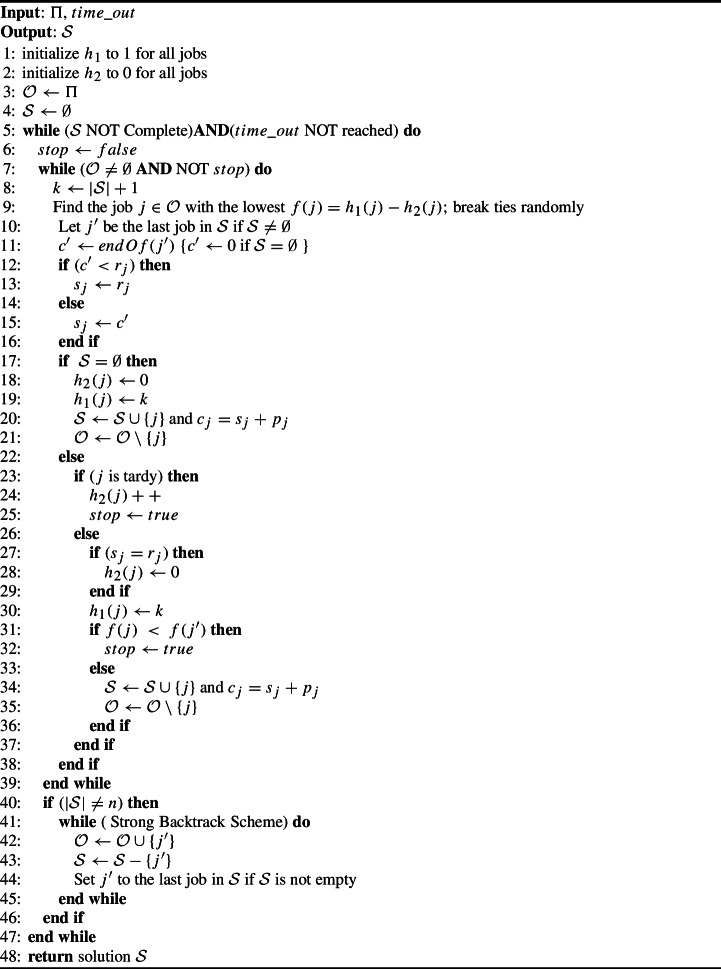
F Search

F defines the set of open jobs to be scheduled: $\mathcal {O}=\Pi$. F stops the construction phase when it either (i) finds a complete feasible schedule (i.e., $|\mathcal {S}|=n$ and $\mathcal {O}=\emptyset$) or (ii) it selects a tardy job. The latter signals that it is impossible to further extend the current partial feasible schedule; thus, triggers the backtracking phase. F chooses the job $j \in \mathcal {O}$ having the smallest *f* value, breaking ties randomly. The random tie breaking makes the search algorithm stochastic. It constitutes the unique stochastic process in the F algorithm.

Depending on $\mathcal {S},$ two cases are possible.

- When $\mathcal {S}$ is empty, *j* is the first selected job; thus, *j* is always on-time and its start time $s_j$ equals its release date $r_j$. This sets $h_2(j)=0$. Therefore, F removes *j* from $\mathcal {O}$ and inserts *j* in $\mathcal {S}$.
- When $\mathcal {S}$ is not empty, *j* is either on-time or tardy.
  - When *j* is tardy, F increments $h_2(j)$ by one and stops the construction phase. Increasing $h_2(j)$ decreases *f*(*j*);  thus, enhances its chances for an earlier selection.
  - When *j* is on-time, F sets $h_2(j)=0$ if *j* starts at its release time and $h_1(j)=k$. Then, two scenarios are possible. In the first scenario, *j* violates Property 2; thus, the construction phase stops. In the second scenario, F appends *j* to $\mathcal {S}$ and removes *j* from $\mathcal {O}$.

The backtracking phase in F iteratively removes jobs from $\mathcal {S}$ and inserts them back to $\mathcal {O}$ until *j* finds a position that satisfies the used backtracking scheme WBS or SBS that are defined above. F then restarts the construction phase selecting job *j*,  which is still in $\mathcal {O}$ and which still has the smallest *f* value among all jobs of $\mathcal {O}.$ Consider, for example, $\mathcal {S}=\{1,2,3,4\},\ f(1)=1,\ f(2)=f(3)=3$ and $f(4)=4.$ If $j=5$ is selected, is on-time, and $f(5)=2,$ F stops the construction phase. Next, F applies the backtracking phase, which removes jobs $4,\ 3,$ and 2 whose *f* values exceed *f*(5). It then returns to the construction phase to successively select jobs $5,\ 1,\ 3$, then 4.

## $\hbox {F}_{I}$: incremental search with F

Despite its good performance, F may require relatively long run times when the number of jobs is very large. To avoid this pitfall, $\hbox {F}_{I}$ augments F with an incremental search; i.e., $\hbox {F}_{I}$ applies a decomposition technique that divides the *n* jobs into *n* branches with branch *k* corresponding to the $k^{th}$ job in the complete input set $\mathcal {O}$. At the $k^{th}$ iteration, $\hbox {F}_{I}$ takes the $k^{th}$ job from $\mathcal {O}$ and inserts it to $\mathcal {Q}$, i.e., the algorithm feeds F with exactly one job, and with the current partial feasible solution $\mathcal {S}$ that already includes $k-1$ jobs. Subsequently, the new task of F is to insert the $k^{th}$ job into the appropriate position in $\mathcal {S}$, so that F obtains a new partial feasible solution with *k* jobs. $\hbox {F}_{I}$ is detailed below.

In iteration 1, $\hbox {F}_{I}$ calls F with $Q=\{1\}$ and $\mathcal {S}=\emptyset .$ The output of F is $\mathcal {S}=\{1\}$ because this single job can be scheduled on time starting at its release time.
In iteration 2, $\hbox {F}_{I}$ calls F with $Q=\{2\}$ and $\mathcal {S}=\{1\}.$ The output is a feasible schedule with jobs 1 and 2 if one exists.
In iteration 3, $\hbox {F}_{I}$ calls F with $Q=\{3\}$ and with the feasible schedule $\mathcal {S}=\{1,2\}$ obtained in iteration 2 and containing jobs 1 and 2 (not necessarily in this order).

Following this pattern, in iteration *n*, F appends job *n* to a feasible schedule of $n-1$ jobs. That is, $\hbox {F}_{I}$ calls F *n* times where each call appends exactly one job $k,\ k=1,\ldots ,n,$ to a partial feasible schedule $\mathcal {S}$. It proceeds as in a merge sort whose objective is to merge *Q* and $\mathcal {S}$.

To call F for the $k^{th}$ time, $\hbox {F}_{I}$ initializes $h_1(k)$ to the next available position $|\mathcal {S}|+1$. It sets $h_2(k)=0$ because *k* was not previously selected. It feeds F with the partial feasible solution stored in $\mathcal {S}$ and with the single job stored in *Q*. Using this input, $\hbox {F}_{I}$ runs Algorithm 2 until it finds a feasible sequence of the *n* jobs or reaches $time\_out$.

Algorithm 2 proceeds as follows. Lines 1–3 initialize $\mathcal {S}=\emptyset ,\ \mathcal {O}=\Pi$ and $Q=\emptyset$. Line 4 sorts $\mathcal {O}$ in a non-decreasing order of the jobs’ deadlines. Lines 5–14 constitute the incremental search, which stops when all jobs in $\mathcal {O}$ are scheduled on-time or the run time exceeds $time\_out$. Lines 6–8 take the job in position 1 from $\mathcal {O}$ and insert it to *Q*. Lines 9–10 assign the next job to position *k* in $\mathcal {S},$ where $k-1=|\mathcal {S}|$ is the current number of scheduled on-time jobs. Line 11 sets $h_2(k)=0$ as *k* was not previously selected. Line 12 calls F, which tries to position the selected job on-time in position *k*. F iterates between the construction and the backtracking phases until it either finds a feasible solution for these inputs or it reaches $time\_out$. Line 15 returns $\mathcal {S},$ which is either a complete or a partially feasible solution.

**Algorithm 2:**
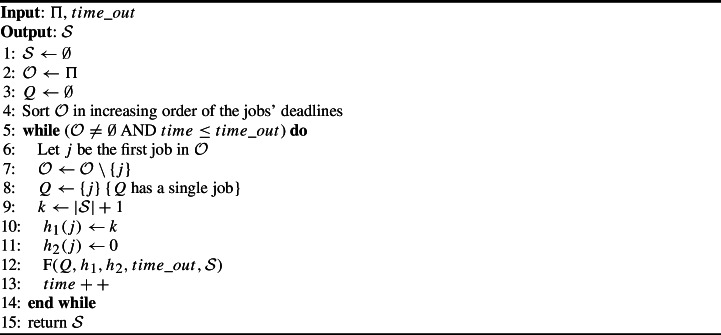
Pseudo code of $\hbox {F}_I$

### Lemma 1

(Correctness) Each iteration of $\hbox {F}_{I}$ generates a partial feasible solution.

### Proof

$\hbox {F}_{I}$ produces partial feasible solutions because, at each iteration, it calls F, which produces feasible solutions. $\square$

## $\hbox {F}_{IR}$: restarts and incremental search with F

$\hbox {F}_{IR}$ restarts $\hbox {F}_{I}$ every time $\hbox {F}_{I}$ fails to identify a feasible solution within run time $time\_out$. This restart strategy allows a good exploration of the state-space and avoids non-promising search directions by halting unsuccessful ones. $\hbox {F}_{IR}$ can’t recognize wrong search directions. It infers, based on its search at a given iteration, that it can’t construct a feasible solution within $time\_out.$ Computational experiments suggest that $time\_out$ can be very small.

As in F, independent restarts of $\hbox {F}_I$ and random tie breaking direct the search toward different partial solutions. Therefore, non-identical solutions may be obtained when $\hbox {F}_I$ is replicated multiple times. Thus, $\hbox {F}_{IR}$ is inherently dotted with a good state-space exploration mechanism because of the stochastic behavior it inherits from F.

Algorithm 3 shows the pseudo-code of $\hbox {F}_{IR}$, which iteratively calls $\hbox {F}_{I}$ (cf. Lines 2–5) until getting a feasible schedule or reaching a maximum number of runs $max\_runs$. Each call has a maximum run time $time\_out$.

**Algorithm 3:**
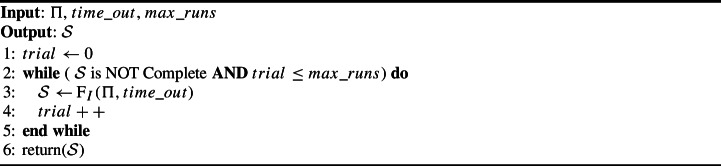
F with Restarts and Incremental Search

### Corollary 1

(Correctness) Each iteration of $\hbox {F}_{IR}$ generates a partial feasible solution.

### Proof

The proof follows directly from Lemma 1. $\square$

## Experimental study

This study assesses the efficiency of the proposed $\hbox {F}_{IR}$ algorithm. Its objective is twofold. First, it highlights the importance of the incremental search and restart strategy in enhancing the performance of F. Second, it shows that $\hbox {F}_{IR}$ solves very large instances with up to a hundred thousand jobs.

In addition to $\hbox {F}_{IR}$, we implemented many algorithms that solve ${\mathfrak {P}}$: SWO, PF, PFX, NEH, and EDD. All algorithms are coded in C++ and run on a desktop with a 2.40 GHz Intel Core i7 processor and a 4.0 GB RAM. To exploit the power of IBM ILOG CP 12.6, we modeled ${\mathfrak {P}}$ using interval variables, noOverlap constraint, and edge-finder propagator.

SWO starts with an initial solution that inserts jobs one-by-one to a position that minimizes the total number of conflicts within the current solution. It then iterates between an analyzer and a prioritizer (improvement) phase. Its analyzer calculates the tardiness of each job. Its prioritizer attempts to move tardy jobs backward giving priority to those having the highest tardiness. Each backward move is coupled with a forward move of one or many non-tardy jobs. SWO stops when it finds a feasible solution or reaches a threshold run time.

PFX is a multiple restart PF. In lieu of starting PF with the longest processing time job, it restarts PF with job $k',\ k'=1,\ldots ,n,$ as the first job, and applies the remaining steps of PF to the remaining $n-1$ jobs.

Herein, NEH, PF and PFX are implemented with three selection criteria: minimal idle time, earliest deadline and earliest completion time. Only the best solution is reported.

EDD is a polynomial time heuristic algorithm that consists of sorting jobs in increasing order of their deadlines. The job with the earliest deadline is the one to be positioned earlier. The use of EDD algorithm in the present experimental study is to measure the number of easy to solve problem instances. It is also used to start the search algorithms F and $\hbox {F}_{IR}$.

### Experimental set up

Performance of implemented algorithms is assessed in terms of run time needed to identify a feasible solution and number of successfully solved instances $\#_s$ over each set. Unless differently stated, a maximal run time of 10 s is allocated for all heuristic approaches F, ACO, LHRTS-DL, SWO, NEH, PF, and PFX, and all run times are in seconds. The number of restarts is set to unlimited for $\hbox {F}_{IR}$ since we assume the feasibility of all instances. The timeout of $\hbox {F}_I$ inside $\hbox {F}_{IR}$ is set to 5 s. If $\hbox {F}_I$ fails to find a feasible solution within 5 s, then $\hbox {F}_{IR}$ stops $\hbox {F}_{I}$ to make a new restart. $\hbox {F}_{IR}$ is replicated 30 times for each instance because of its random nature. Then, the minimal ($RT_{\ell }$), average (*RT*), maximal ($RT^u$) and standard deviation ($\sigma _{RT}$) of the 30 run times are computed. Finally, all comparisons are inferred based on statistical testing at a 5% significance level.

The algorithms are applied to a large data set of generated instances whose difficulty is a function of their size *n*,  disjunctive ratio, similarity ratio, machine availability horizon of length $d_{n},$ and machine’s utilization *U*. The disjunctive ratio reflects the ratio of the number of conflicting pairs of jobs (i.e., whose time windows overlap) to the total number of pairs of distinct jobs (Baptiste et al., 2001). (Two distinct jobs *A* and *B* are said to be conflicting if the fact of scheduling *A* before *B* results in the tardiness of *B*.) The similarity ratio reflects the homogeneity of the jobs. It is the ratio of the number of jobs having large processing times to the number of those having short processing times. Finally, *U* is the ratio of the sum of the processing times to the length of the time horizon: $U = \frac{1}{d_{n}} \sum _{j \in \Pi } {p_j}.$ It reflects the density or sparsity of the instance. The larger *U* is, the busier the machine. When $U=1.00,$ the machine has no idle time during $[0,d_{n}]$.

All the aforementioned five factors are taken into account during the generation of the data sets either explicitly or implicitly. That is, the similarity index is reflected by the standard deviation $\sigma$ of the processing times of the jobs. When $\sigma$ is small (resp. large), the jobs are homogeneous (resp. heterogeneous). The disjunctive ratio is reflected by the width of the time windows of the jobs. The larger the time windows, the lower the disjunctive ratio. The width of any time window is a function of a pre-specified parameter *TW*.

This experimental investigation considers two types of instances: pseudo random and random. While pseudo random instances (of a given data set) inherit parts of the previous instances and build on them, random ones are independent. All instances are available upon request.

#### Pseudo random instances

The pseudo-random generator uses an initial instance $\hbox {I}_1$ having a given number of jobs and a scheduling horizon $d_{n}$ with a known feasible solution $\mathcal {S}_1$ and a list of idle time windows $\mathcal {I}_1$. It builds new instances out of $\hbox {I}_1$ by filling the idle times of $\mathcal {I}_1$ with additional jobs. It adds these jobs sequentially as follows. It chooses, randomly, an idle time window $\iota =[b_{\ell },b^u] \in \mathcal {I}_1,$ where $b_{\ell }$ and $b^u$ are respectively the lower and the upper bounds of $\iota .$ It then selects a processing time $p_j$ for the job *j* to be added. It initializes a parameter $\alpha =6,$ and sets $p_j=\lfloor \frac{b^u-b_{\ell }}{\alpha }\rfloor$ if $b^u-b_{\ell } > \alpha ;$ otherwise, it decreases $\alpha$ by one and rechecks whether the new condition holds. It repeats this process until either it fixes $p_j$ or $\alpha$ becomes less than two. Having fixed $p_j,$ the generator selects a release date $r_j$ from the discrete uniform$[0,b_{\ell }]$, and sets the deadline $d_j=\lfloor b_{\ell }+\beta p_j\rfloor ,$ where $\beta$ is a random number from the continuous uniform[2, 3]. The generator keeps adding jobs until it obtains an instance whose number of jobs $n \in [n_{\ell },n^u]$ and load $U \in [U_{\ell },U^u].$ The lower and upper bounds $n_{\ell },\ n^u,\ U_{\ell },\ U^u$ of *n* and *U* are fixed for each data set.

The generator obtained 100 feasible instances using an instance $\hbox {I}_1$ with $d_{n}=500$ and 26 initial jobs for small-sized instances. For medium sized instances, it used real-world instance $\hbox {I}_1$ from real-time systems (Cavalcante, 1997) with $d_{n}=30,000$ and 781 initial jobs.

#### Random instances

The random instances are grouped into data sets that correspond to combinations of various levels of $n,\ U,\ \mu _p,\ \rho _p,$ and $TW.\ \ \mu _p$ and $\rho _p$ are the mean and coefficient of variation of the processing times of the *n* jobs. The relative range of release times *R* and the relative range of deadlines *D* are drawn from a discrete uniform [0, *TW*].

The random generator draws *n* processing times from the Normal$(\mu _p,\sigma _p)$ rounded to the nearest integer. It sorts the jobs randomly, and computes for each job $j,\ j \in \Pi ,$ its starting time $S_j$ and completion time $C_j.$ Consequently, it calculates the release time $r_j=S_j-R$ and the deadline $d_j = C_j+D$. For each data set, it generates 30 instances.

For medium-sized instances, the generator produces 128 data sets (thus a total of 3840 instances) corresponding to $n=1500,\ 2000,\ 2500,\ 3000,\ U=0.6,\ 0.7,\ 0.8,\ 0.9,$ with an error $\bigtriangleup =0.025,\ \mu _p=50,\ 500,\ \rho _p= 4,\ 50,$ and $TW=2\mu _p, 500\mu _p.$ This set is particularly very hard. It spans a large panoply of problem types: short versus large and homogeneous versus highly heterogeneous processing times, under versus over utilization of the machine, and high versus low disjunctive ratios.

For large-sized instances, the generator produces 3840 instances, classified into 128 data sets. The sets correspond to $n=25,000,\ 50,000,\ 75,000,\ 100,000$, $U=0.6,\ 0.7,\ 0.8,\ 0.9,$ with an error $\bigtriangleup =0.025,\ \mu _p=50,\ 500,\ \rho _p= 4, 50,$ and $TW=2\mu _p,\ 4\mu _p.$

### Performance of $\hbox {F}_{IR}$ on medium-sized instances

None of the 100 pseudo-random instances and 3840 random instances is solvable using CPLEX, ACO, LHRTS-DL, SWO, NEH, or F. Therefore, Table 1 reports the number of instances solved by PF, PFX, $\hbox {F}_{IR}$, and the state-of-that-art CSP solver ILOG CP with an allocated run time of 2700 s; thus, the column label $\hbox {CP}_{2700}$. Both PF and PFX fail to solve the 100 pseudo-random instances of the medium-sized pool and more than half of the random ones; thus, are discarded in future comparisons. On the other hand, $\hbox {F}_{IR}$ solves all instances, be them pseudo or pure random. Pseudo-random instances are challenging for ILOG CP, which fails to solve any of them within 2700 s. However, ILOG CP succeeds to solve all random instances. Sections 6.2.1 and 6.2.2 detail the results for both data sets.

**Table 1:** Number of medium-sized instances solved by PF, PFX, $\hbox {F}_{IR}$, and $\hbox {CP}_{2700}$

		$$\#_s$$			
Instance type	# of instances	PF	PFX	$\hbox {F}_{IR}$	$\hbox {CP}_{2700}$
random	3840	1917	1918	**3840**	**3840**
pseudo-random	100	0	0	**100**	0

Bold values indicate that the method solved all instances

#### Results on random instances

Table 2 reports statistics of the observed run time of ILOG CP and $\hbox {F}_{IR}$ for each set. Columns 1–5 indicate the characteristics of the problems: $n,\ U,\ \mu _p,\ \rho ,$ and *TW*. Columns 6 and 11 display the average runtime $RT_{CP}$ of the ILOG CP solver. Finally, Columns 7–10 and 12–15 report respectively ${RT}_{\ell },\ RT,\ {RT}^u,$ and $\sigma _{RT},$ which are computed over all replications of each set.

**Table 2:** Statistics of the runtime of ILOG CP and $\hbox {F}_{IR}$ on medium-sized instances

*n*	U	$\mu _p$	$\rho$	*TW*	$RT_{CP}$	${RT}_{\ell }$	*RT*	${RT}^u$	$\sigma _{RT}$	*n*	$RT_{CP}$	${RT}_{\ell }$	*RT*	${RT}^u$	$\sigma _{RT}$
1500	0.6	50	4	100	12.6057	0.0019	0.0021	0.0048	0.0002	2500	59.4180	0.0045	0.0052	0.0091	0.0003
				25,000	3.1757	0.0019	0.0021	0.0039	0.0002		13.5200	0.0063	0.0105	0.0258	0.0027
			50	100	10.3570	0.0019	0.0022	0.0048	0.0002		48.7130	0.0064	0.0099	0.0121	0.0005
				25,000	3.0223	0.0019	0.0032	0.0563	0.0056		13.5493	0.0063	0.0097	0.0123	0.0004
		500	4	1000	12.8063	0.0019	0.0020	0.0043	0.0001		60.0720	0.0045	0.0053	0.0109	0.0001
				250,000	3.0917	0.0019	0.0020	0.0036	0.0000		14.0593	0.0045	0.0052	0.0073	0.0001
			50	1000	10.0887	0.0019	0.0021	0.0045	0.0002		47.4680	0.0064	0.0100	0.0124	0.0004
				250,000	2.9897	0.0019	0.0020	0.0037	0.0001		13.8173	0.0057	0.0117	0.0434	0.0069
	0.7	50	4	100	14.3347	0.0019	0.0021	0.0033	0.0000		66.0827	0.0046	0.0075	0.0124	0.0022
				25,000	3.2710	0.0019	0.0024	0.0237	0.0021		14.4463	0.0061	0.0096	0.0122	0.0005
			50	100	12.3867	0.0019	0.0022	0.0044	0.0002		58.3933	0.0065	0.0101	0.0126	0.0005
				25,000	3.0657	0.0019	0.0023	0.0111	0.0009		13.7360	0.0064	0.0102	0.0220	0.0017
		500	4	1000	13.9973	0.0019	0.0021	0.0038	0.0001		66.8193	0.0046	0.0053	0.0077	0.0001
				250,000	3.7603	0.0019	0.0041	0.0924	0.0113		15.9140	0.0045	0.0070	0.0234	0.0048
			50	1000	12.0970	0.0020	0.0021	0.0039	0.0001		57.2480	0.0068	0.0102	0.0124	0.0004
				250,000	3.1003	0.0019	0.0020	0.0039	0.0000		13.9550	0.0061	0.0104	0.0253	0.0020
	0.8	50	4	100	14.7823	0.0020	0.0022	0.0035	0.0001		68.4487	0.0069	0.0106	0.0130	0.0005
				25,000	4.4063	0.0019	0.0021	0.0048	0.0002		35.7970	0.0065	0.0251	0.1467	0.0308
			50	100	13.7097	0.0020	0.0023	0.0047	0.0002		64.2663	0.0061	0.0105	0.0133	0.0005
				25,000	3.6040	0.0019	0.0031	0.0456	0.0053		18.7740	0.0053	0.0180	0.1224	0.0240
		500	4	1000	14.5703	0.0020	0.0022	0.0037	0.0001		69.5747	0.0046	0.0053	0.0076	0.0001
				250,000	5.2727	0.0019	0.0308	0.2961	0.0650		20.6097	0.0045	0.0085	0.0664	0.0116
			50	1000	13.4883	0.0020	0.0021	0.0038	0.0001		62.9780	0.0065	0.0106	0.0129	0.0005
				250,000	4.7533	0.0019	0.0230	0.3889	0.0739		16.0113	0.0053	0.0113	0.0415	0.0057
	0.9	50	4	100	13.9427	0.0023	0.0030	0.0091	0.0006		63.6070	0.0080	0.0134	0.0198	0.0018
				25,000	18.0687	4.9181	38.2452	95.4587	7.2485		79.3757	0.0047	1.0349	20.7872	1.4764
			50	100	13.7380	0.0023	0.0033	0.0101	0.0008		63.6687	0.0083	0.0131	0.0252	0.0019
				25,000	13.5847	7.5031	41.6388	113.0960	8.4352		63.5187	0.0069	5.5990	304.7700	15.9360
		500	4	1000	14.2013	0.0022	0.0027	0.0052	0.0003		66.4650	0.0049	0.0062	0.0090	0.0004
				250,000	14.2947	8.1508	44.9639	114.6090	9.9385		83.8250	0.0629	139.7674	526.7370	128.1057
			50	1000	13.5723	0.0021	0.0026	0.0054	0.0003		64.6143	0.0074	0.0121	0.0165	0.0008
				250,000	15.7537	14.5694	47.7363	96.5040	7.0228		56.6800	0.7880	108.0957	521.5670	96.2821
2000	0.6	50	4	100	30.4923	0.0031	0.0034	0.0051	0.0000	3000	105.6303	0.0062	0.0067	0.0150	0.0002
				25,000	6.9380	0.0030	0.0033	0.0056	0.0000		22.9440	0.0061	0.0067	0.0134	0.0003
			50	100	24.4767	0.0034	0.0067	0.0083	0.0002		77.3833	0.0062	0.0067	0.0135	0.0004
				25,000	6.8577	0.0041	0.0078	0.0525	0.0073		22.8620	0.0061	0.0068	0.0146	0.0005
		500	4	1000	29.8160	0.0031	0.0034	0.0052	0.0000		103.3643	0.0062	0.0068	0.0152	0.0006
				250,000	7.1440	0.0044	0.0068	0.0145	0.0012		23.4027	0.0061	0.0066	0.0137	0.0003
			50	1000	23.2263	0.0038	0.0067	0.0086	0.0003		81.4827	0.0062	0.0069	0.0145	0.0005
				250,000	6.8677	0.0042	0.0064	0.0084	0.0003		23.6010	0.0061	0.0070	0.0166	0.0015
	0.7	50	4	100	34.1280	0.0031	0.0034	0.0056	0.0001		106.3230	0.0062	0.0068	0.0150	0.0002
				25,000	7.1687	0.0030	0.0033	0.0056	0.0001		24.3870	0.0061	0.0097	0.1117	0.0125
			50	100	29.5170	0.0044	0.0068	0.0092	0.0004		94.9283	0.0062	0.0068	0.0139	0.0003
				25,000	7.0763	0.0041	0.0065	0.0088	0.0004		23.3790	0.0061	0.0071	0.0351	0.0027
		500	4	1000	33.3680	0.0031	0.0040	0.0091	0.0007		113.2643	0.0062	0.0069	0.0151	0.0005
				250,000	7.6643	0.0042	0.0066	0.0090	0.0003		25.0563	0.0061	0.0075	0.0394	0.0040
			50	1000	28.7083	0.0046	0.0067	0.0087	0.0003		98.1233	0.0062	0.0068	0.0150	0.0004
				250,000	7.2720	0.0046	0.0082	0.0530	0.0070		23.9983	0.0061	0.0089	0.0784	0.0090
	0.8	50	4	100	35.0380	0.0032	0.0036	0.0055	0.0001		109.3617	0.0064	0.0071	0.0156	0.0003
				25,000	8.9423	0.0030	0.0043	0.0212	0.0030		29.1293	0.0061	0.0141	0.1164	0.0209
			50	100	32.8053	0.0045	0.0069	0.0093	0.0003		111.0140	0.0063	0.0070	0.0152	0.0002
				25,000	8.1293	0.0044	0.0106	0.0975	0.0165		31.9207	0.0061	0.0162	0.1876	0.0305
		500	4	1000	34.4487	0.0033	0.0068	0.0090	0.0009		120.1780	0.0063	0.0069	0.0150	0.0003
				250,000	10.1227	0.0045	0.0083	0.0617	0.0096		35.0673	0.0061	0.0165	0.1294	0.0253
			50	1000	31.8640	0.0047	0.0069	0.0089	0.0004		110.0663	0.0063	0.0070	0.0151	0.0004
				250,000	7.8707	0.0046	0.0072	0.0275	0.0031		25.6437	0.0061	0.0094	0.0964	0.0146
	0.9	50	4	100	32.9240	0.0037	0.0049	0.0091	0.0008		104.9663	0.0071	0.0091	0.0244	0.0015
				25,000	46.2143	1.3114	70.8268	278.2060	40.4143		144.6927	0.0061	0.0546	0.5195	0.0810
			50	100	32.1430	0.0053	0.0089	0.0150	0.0012		111.3780	0.0067	0.0084	0.0190	0.0011
				25,000	26.5577	1.9652	73.6292	244.1290	34.4695		82.4933	0.0061	0.0853	1.5287	0.1751
		500	4	1000	33.2267	0.0052	0.0087	0.0136	0.0010		115.9027	0.0068	0.0083	0.0197	0.0008
				250,000	51.4413	9.2952	138.7209	282.4940	27.3366		111.9700	0.0748	20.9387	832.0430	90.1710
			50	1000	32.4513	0.0043	0.0085	0.0124	0.0010		111.2490	0.0068	0.0082	0.0202	0.0009
				250,000	36.4013	7.8959	138.6511	284.4710	25.5070		114.7550	0.0648	3.3591	93.2212	6.5953

**Fig. 1:**
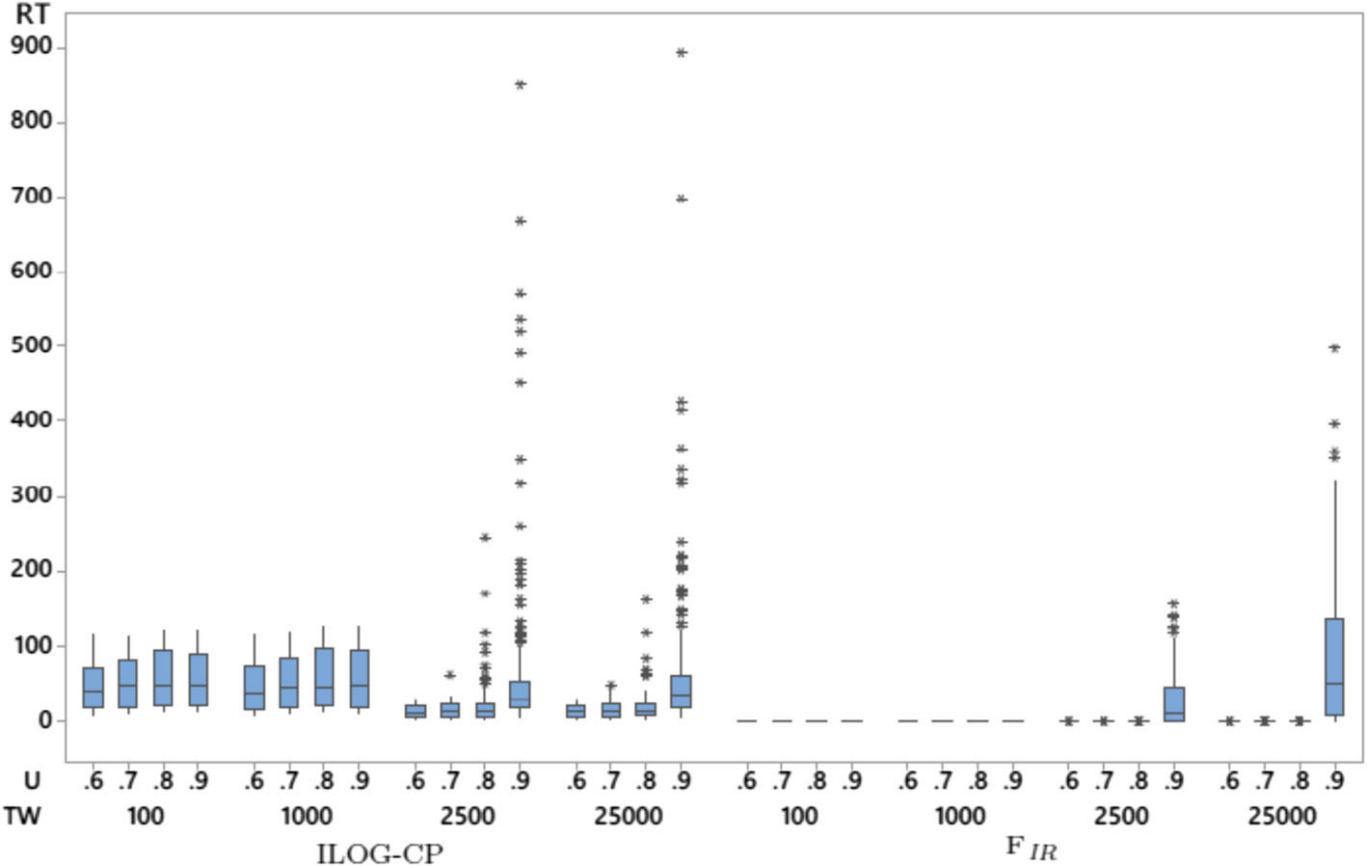
Box plot of the run times of ILOG CP and $\hbox {F}_{IR}$ as a function of the machine’s utilization and range of time windows

$\hbox {F}_{IR}$ solves every instance of the random data-sets. It solves hard instances with 3000 jobs in less than one minute (except for one outlier that required 497.008 s). In fact, 75% of the instances are solved in less than 0.008 s. $\hbox {F}_{IR}$ encounters some outlier cases when $U=0.9$ and $TW=2500$ or 25000 corresponding to highly loaded machines and wide time windows. This is further confirmed by Fig. 1 which displays the box plots of the run times of ILOG CP and $\hbox {F}_{IR}$ as a function of *U* and *TW*. Even though ILOG CP with its edge-finding propagator solves all instances too, its mean runtime is larger than the mean runtime of $\hbox {F}_{IR}$ at any level of significance with a mean difference point estimate of 32.10 s and a 95% lower bound of 30.60 s. Therefore, ILOG CP is not further discussed.

#### Results on Pseudo-random instances

Table 3 reports statistics on the number of restarts that $\hbox {F}_{IR}$ required to find a feasible solution when replicated 100 times for each of the pseudo random instances, with $\hbox {F}_{I}$ allocated 3 s. Columns 1–3 and 8–10 show the instance *I*, its size *n*, and its utilization *U*. Columns 4–7 and 11–14 display ${\bar{\eta }},\ {\underline{\eta }},\ {\eta },$ and $\sigma _{\eta },$ the maximum, minimum, average, and standard deviation of the number of restarts $\hbox {F}_{IR}$ needed to solve the instance. Table 3 suggests the following.

- The largest number of restarts needed to solve any instance is very small except for few outliers such as ${\bar{\eta }}=67$ for I41. These outliers remain, however, very small compared to the problem size. In fact, the largest run time over all replications over all instances is less than 4 min whereas the run time of IBM ILOG CP ranges between 18 and 50 h.
- Many instances do not require a restart (i.e., have ${\underline{\eta }}=1$). This emphasizes the importance of the incremental search in enhancing the performance of F, which doesn’t solve any instance.
- The number of restarts over all instances averages 5.85; yielding an 18 s point estimate of the mean run time of $\hbox {F}_{IR}$. This very reduced run time per instance highlights the importance of the restart strategy in diversifying the search and identifying a feasible solution.
- $\sigma _{\eta }$ averages 4.87 over all tested instances; yielding a less than 20 s confidence interval upper bound estimate of the mean run time of $\hbox {F}_{IR}$. The small magnitude of $\sigma _{\eta }$ further suggests the robustness of $\hbox {F}_{IR}$.

**Table 3:** Statistics on the number of restarts of $\hbox {F}_{IR}$

*Instance*	*n*	*U*	${\bar{\eta }}$	${\underline{\eta }}$	${\eta }$	$\sigma _{\eta }$	*Instance*	*n*	*U*	${\bar{\eta }}$	${\underline{\eta }}$	${\eta }$	$\sigma _{\eta }$
I1	7127	0.850	21	2	9	7	I51	7177	0.852	21	1	6	6
I2	7128	0.850	19	2	7	5	I52	7178	0.852	19	1	10	7
I3	7129	0.850	8	1	3	2	I53	7179	0.852	10	1	3	3
I4	7130	0.850	6	1	2	2	I54	7180	0.852	4	1	2	1
I5	7131	0.850	9	1	5	3	I55	7181	0.852	14	1	5	4
I6	7132	0.850	10	1	4	3	I56	7182	0.852	6	1	3	2
I7	7133	0.850	6	1	3	2	I57	7183	0.853	6	1	2	2
I8	7134	0.850	6	1	2	2	I58	7184	0.853	7	1	3	2
I9	7135	0.850	4	1	2	1	I59	7185	0.853	7	1	3	2
I10	7136	0.850	4	1	2	1	I60	7186	0.853	4	1	2	1
I11	7137	0.850	12	1	4	4	I61	7187	0.853	3	1	2	1
I12	7138	0.850	9	1	4	3	I62	7188	0.853	4	1	2	1
I13	7139	0.850	10	1	5	3	I63	7189	0.853	7	1	3	2
I14	7140	0.851	14	2	5	4	I64	7190	0.853	5	1	3	1
I15	7141	0.851	15	1	7	4	I65	7191	0.853	6	1	2	2
I16	7142	0.851	18	1	7	6	I66	7192	0.853	3	1	1	1
I17	7143	0.851	25	2	12	7	I67	7193	0.853	2	1	2	1
I18	7144	0.851	22	1	6	6	I68	7194	0.853	3	1	2	1
I19	7145	0.851	16	1	7	6	I69	7195	0.853	4	1	3	1
I20	7146	0.851	10	1	4	4	I70	7196	0.853	3	1	2	1
I21	7147	0.851	17	1	5	5	I71	7197	0.853	4	1	2	1
I22	7148	0.851	23	1	6	7	I72	7198	0.853	18	1	5	5
I23	7149	0.851	16	1	5	4	I73	7199	0.853	20	1	5	6
I24	7150	0.851	10	1	4	3	I74	7200	0.853	10	1	3	3
I25	7151	0.851	11	1	3	3	I75	7201	0.853	7	1	2	2
I26	7152	0.851	14	1	5	4	I76	7202	0.854	12	1	5	4
I27	7153	0.851	12	1	5	4	I77	7203	0.854	19	1	9	7
I28	7154	0.851	8	1	3	3	I78	7204	0.854	48	2	11	13
I29	7155	0.851	7	1	2	2	I79	7205	0.854	31	1	15	10
I30	7156	0.851	4	1	2	1	I80	7206	0.854	23	1	6	6
I31	7157	0.851	7	1	2	2	I81	7207	0.854	39	5	15	10
I32	7158	0.851	7	1	2	2	I82	7208	0.854	12	2	6	3
I33	7159	0.851	5	1	2	1	I83	7209	0.854	18	1	6	6
I34	7160	0.852	8	1	4	2	I84	7210	0.854	28	2	9	8
I35	7161	0.852	3	1	2	1	I85	7211	0.854	18	1	5	5
I36	7162	0.852	4	1	2	1	I86	7212	0.854	17	2	6	4
I37	7163	0.852	5	1	2	1	I87	7213	0.854	24	1	7	7
I38	7164	0.852	5	1	2	1	I88	7214	0.854	30	2	10	11
I39	7165	0.852	31	1	9	9	I89	7215	0.854	24	1	11	7
I40	7166	0.852	23	2	11	8	I90	7216	0.854	13	1	4	4
I41	7167	0.852	67	3	18	23	I91	7217	0.854	11	1	5	4
I42	7168	0.852	36	3	14	12	I92	7218	0.854	11	1	5	3
I43	7169	0.852	26	2	10	7	I93	7219	0.854	21	1	7	8
I44	7170	0.852	43	2	13	14	I94	7220	0.854	12	3	7	4
I45	7171	0.852	27	2	9	8	I95	7221	0.854	16	1	6	4
I46	7172	0.852	46	1	14	16	I96	7222	0.854	26	1	8	8
I47	7173	0.852	22	1	9	7	I97	7223	0.854	33	1	10	10
I48	7174	0.852	26	1	10	8	I98	7224	0.855	18	1	6	6
I49	7175	0.852	50	1	19	16	I99	7225	0.855	19	1	9	7
I50	7176	0.852	36	1	17	13	I100	7226	0.855	21	1	8	6

**Table 4:** Runtime of IBM ILOG CP

Instance	n	Time (seconds)
I1	7127	122,353
I2	7128	179,349
I3	7129	66,108
I4	7130	179,224
I5	7131	180,291
I6	7132	179,845
I7	7133	167,914
I8	7134	66,678
I9	7135	168,308
I10	7136	179,773

Thanks to its incremental search and restart strategy, $\hbox {F}_{IR}$ solves all medium sized instances within a reduced run time. IBM ILOG CP solves all these instances too but within a very large run time that is impractical for real life instances. Table 3 shows that $\hbox {F}_{IR}$ solves the pseudo random instances within a small and reasonable run time. Solving them via the IBM ILOG-CP solver requires excessively long run times as Table 4 exemplifies for the first ten instances of this set. Instances with a low disjunctive ratio (0.277) are challenging for CSP solvers. The small number of conflicting pairs of jobs does not fathom large portions of the search space. Thus, the solver needs to investigate numerous search directions before finding a ‘correct’order of jobs.

### Performance of $\hbox {F}_{IR}$ on large instances

When applied to large-sized instances with up to 100,000 jobs, $\hbox {F}_{IR}$ has a 100% success rate. Therefore, Table 5 reports only the run time. Columns 1–4 indicate the characteristics of the problems: $n,\ U,\ \mu _p,$ and $\rho .$ Columns 5–8 and 9–12 report ${RT}_{\ell },\ RT,\ {RT}^u,$ and $\sigma _{RT}$ for $TW=2\mu _p$ and $4\mu _p$, respectively. For these instances, the statistics of the number of restarts over 30 replications of each instance reveal little variability and very low values: $\eta _{\ell }= \eta =\eta ^u=1,$ for $n=25,000$ and 50, 000; $\eta _{\ell }= \eta =2$ and $\eta ^u=3,$ for $n=75,000$; and $\eta _{\ell }= \eta =4$ and $\eta ^u=6,$ for $n=100,000$. Therefore, the number of restarts is not further discussed.

**Fig. 2:**
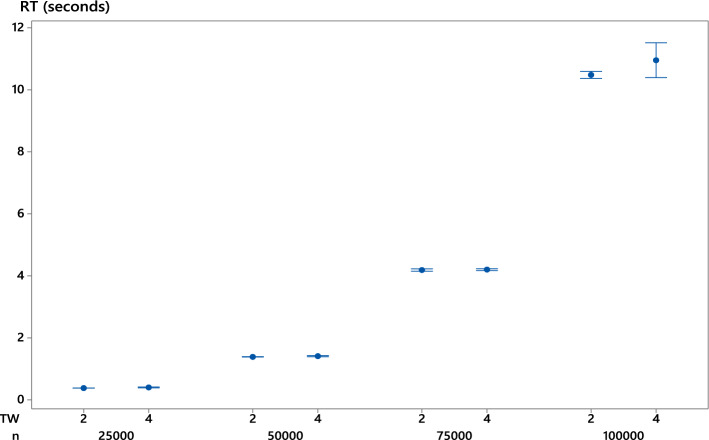
95% confidence intervals of the mean run times of $\hbox {F}_{IR}$ as a function of instance size *n* and time windows *TW*

Table 5 shows that the run time of $\hbox {F}_{IR}$ is very small despite the large size of the instances. Its average is of the order of 10 s for $n=$ 100,000 jobs. This performance is independent of $U,\ \mu _p,\ \rho _p,$ and *TW* at any level of significance. However, the mean run time of $\hbox {F}_{IR}$ increases as *n* increases, most likely because of a larger number of restarts. Figure 2 further supports the above findings. It displays the 95% confidence intervals of the mean run time as a function of *n* and *TW*.

**Table 5:** Run time of $\hbox {F}_{IR}$ on large-sized instances

				$TW=2\mu _p$				$TW=4\mu _p$			
*n*	*U*	$\mu _p$	$\rho _p$	${RT}_{\ell }$	*RT*	${RT}^u$	$\sigma _{RT}$	${RT}_{\ell }$	*RT*	${RT}^u$	$\sigma _{RT}$
25,000	0.6	50	4	0.3138	0.3753	0.8617	0.0169	0.3162	0.3718	0.3974	0.0255
			50	0.3123	0.3756	0.4584	0.0037	0.3172	0.4354	0.4124	0.0255
		500	4	0.3164	0.3769	0.4629	0.0033	0.3205	0.4173	0.4213	0.0248
			50	0.3128	0.3757	0.4538	0.0055	0.3516	0.4496	0.4522	0.0194
	0.7	50	4	0.3149	0.3774	0.9194	0.0119	0.3124	0.3839	0.4026	0.0197
			50	0.3142	0.3763	0.4335	0.0037	0.3137	0.3841	0.4012	0.0206
		500	4	0.3139	0.3770	0.4044	0.0045	0.3189	0.3891	0.4118	0.0196
			50	0.3137	0.3779	0.4070	0.0033	0.3487	0.4232	0.4421	0.0193
	0.8	50	4	0.3170	0.3802	0.4226	0.0027	0.3130	0.3868	0.4017	0.0219
			50	0.3155	0.3781	0.4131	0.0043	0.3152	0.3865	0.4060	0.0210
		500	4	0.3161	0.3743	0.9560	0.0190	0.3203	0.3937	0.4141	0.0213
			50	0.3154	0.3799	0.4157	0.0035	0.3427	0.4252	0.4393	0.0205
	0.9	50	4	0.3262	0.3913	0.4337	0.0060	0.3150	0.3826	0.4017	0.0214
			50	0.3234	0.3887	0.4368	0.0042	0.3160	0.3843	0.4047	0.0217
		500	4	0.3239	0.3875	0.4665	0.0048	0.3182	0.3880	0.4081	0.0220
			50	0.3220	0.3865	0.4830	0.0040	0.3376	0.4094	0.4278	0.0229
50,000	0.6	50	4	1.2809	1.3988	4.3574	0.0341	1.2989	1.3834	1.4263	0.0235
			50	1.2830	1.3728	1.4761	0.0092	1.2937	1.3929	1.4257	0.0230
		500	4	1.2946	1.3799	1.5007	0.0132	1.3014	1.4024	1.4425	0.0260
			50	1.2963	1.3874	1.5066	0.0152	1.3466	1.5140	1.5225	0.0264
	0.7	50	4	1.2872	1.3816	1.5237	0.0114	1.2985	1.3870	1.4330	0.0240
			50	1.2779	1.3827	1.4947	0.0120	1.3015	1.4065	1.4521	0.0296
		500	4	1.2791	1.3762	1.4925	0.0118	1.3026	1.4112	1.4496	0.0266
			50	1.2877	1.3660	1.4549	0.0088	1.3651	1.4522	1.5026	0.0252
	0.8	50	4	1.2785	1.3854	1.4774	0.0077	1.2937	1.3905	1.4345	0.0251
			50	1.2938	1.3863	1.4975	0.0109	1.2946	1.3891	1.4225	0.0233
		500	4	1.2815	1.3736	1.4749	0.0071	1.2990	1.3890	1.4339	0.0217
			50	1.2745	1.3859	1.5016	0.0124	1.3544	1.4442	1.4959	0.0244
	0.9	50	4	1.3148	1.3988	1.5013	0.0121	1.2827	1.3868	1.4427	0.0287
			50	1.3182	1.3946	1.5072	0.0088	1.3003	1.3816	1.4228	0.0221
		500	4	1.3065	1.4045	1.4982	0.0075	1.2893	1.3918	1.4377	0.0259
			50	1.3126	1.3998	1.5185	0.0120	1.3469	1.4453	1.4776	0.0247
75,000	0.6	50	4	3.9901	4.1512	7.5648	0.0269	3.9731	4.1598	4.2452	0.0472
			50	3.9844	4.1509	4.4370	0.0211	4.0042	4.1642	4.2505	0.0479
		500	4	3.9888	4.1481	4.4611	0.0185	4.0063	4.1596	4.2466	0.0446
			50	4.0032	4.1782	4.4139	0.0254	4.1163	4.3400	4.4089	0.0543
	0.7	50	4	3.9940	4.1474	4.4550	0.0223	3.9903	4.2572	4.3154	0.0592
			50	3.9803	4.1549	4.4248	0.0227	3.9822	4.1570	4.2623	0.0519
		500	4	3.9932	4.1632	4.7832	0.0165	4.0044	4.1927	4.2756	0.0502
			50	3.9909	4.1763	4.4042	0.0210	4.0834	4.2513	4.3451	0.0475
	0.8	50	4	3.9881	4.1799	12.2821	0.0780	4.0110	4.1533	4.2629	0.0503
			50	4.0002	4.1654	4.4573	0.0254	4.0045	4.1499	4.2293	0.0449
		500	4	3.9759	4.1561	4.4210	0.0195	4.0172	4.1760	4.2627	0.0486
			50	4.0032	4.1836	4.4528	0.0179	4.0987	4.2619	4.3684	0.0560
	0.9	50	4	4.0319	4.2001	4.6751	0.0266	4.0111	4.1845	4.3038	0.0584
			50	4.0182	4.1852	4.4461	0.0165	4.0046	4.1993	4.3182	0.0669
		500	4	4.0178	4.2047	4.4764	0.0242	3.9972	4.1669	4.2750	0.0516
			50	4.0416	4.4379	30.4288	1.1802	4.0666	4.2442	4.3682	0.0548
100,000	0.6	50	4	9.6073	10.2744	15.0956	0.2736	9.5965	10.2937	12.3641	0.6017
			50	9.6124	10.1875	15.2574	0.2999	9.5872	9.9728	11.2258	0.2917
		500	4	9.6423	10.3533	15.2121	0.4448	9.5830	10.4851	13.2197	0.7452
			50	9.6461	10.4106	15.1832	0.3674	9.7357	14.0317	15.0497	1.8890
	0.7	50	4	9.6266	10.3468	15.1982	0.2661	9.6052	10.1269	12.0327	0.4833
			50	9.6245	10.3516	15.3412	0.2956	9.5986	10.3052	11.3831	0.3509
		500	4	9.6242	10.5433	15.2392	0.4173	9.5797	10.4990	11.7440	0.5081
			50	9.6493	10.3053	15.1475	0.3131	9.7513	11.3993	14.6474	1.5058
	0.8	50	4	9.6394	10.4117	15.2667	0.3378	9.5834	10.4856	11.3926	0.3827
			50	9.6115	10.5551	15.1158	0.3254	9.5741	10.5017	11.7426	0.4332
		500	4	9.6384	10.4244	15.1905	0.3873	9.6417	10.7170	14.4208	1.2216
			50	9.6537	10.3936	15.2675	0.3460	9.6853	12.6110	15.4012	1.8632
	0.9	50	4	9.6640	10.9073	15.4886	0.5928	9.6006	10.3217	11.7383	0.4453
			50	9.6047	10.8795	15.3431	0.4547	9.5819	10.8521	13.5703	0.8707
		500	4	9.6545	10.5291	15.2163	0.4447	9.6194	11.0298	13.6002	0.9294
			50	9.6869	10.7957	16.6547	0.7619	9.7242	11.5977	14.5246	1.5183

### Discussion

Table 6 summarizes the performance of all implemented algorithms and of IBM ILOG CP on all types of small and medium sized instances with results of F, ACO, and LHRTS-DL taken form Laalaoui and M’Hallah (2022). Clearly, $\hbox {F}_{IR}$ outperforms all other approaches. It is the unique approach that solves almost all instances within 2700 s, and is scalable for large instances as Table 5 shows. IBM ILOG CP is the second best approach but its performance degrades for medium-sized instances. It is very sensitive to the size of the instance; thus, is not well suited for large-sized real-life instances.

In terms of run time, $F_{IR}$, which has a stable run time, outperforms all other approaches. When the lengths of time windows are relatively small, $F_{IR}$ finds feasible solutions instantly (cf. Figs. 1 and 2). Larger time windows are more difficult because of their small disjunction ratios, which increase the number of non-conflicting pairs of jobs and make $\hbox {F}_{IR}$ use more time to decide the order of jobs in the final feasible solution.

Finally, $\hbox {F}_{IR}$ is the unique approach that solves very large problem instances with up to 100,000 jobs within a minute. To the best of the authors’ knowledge, no other approach scales to this problem size.

**Table 6:** Number of solved instances on small and medium sized instances

		#instances	NEH	SWO	ACO	LHRTS-DL	F	$\hbox {F}_{EDD}$	$\hbox {F}_{100}$	$\hbox {F}_{IR}(2700)$	$\hbox {CP}_{2700}$	PF	PFX
Small	Random	480	83	304	288	303	**480**	**480**	**480**	**480**	**480**	**480**	**480**
	Pseudo	2000	0	–	874	791	1298	1615	1614	1996	**2000**	355	508
Medium	Random	3840	0	–	–	–	–	–	–	**3840**	**3840**	1917	1918
	Pseudo	100	0	–	–	–	–	–	–	**100**	0	0	0

Bold values indicate that the method solved all instances

## Conclusion

This paper considers the decision problem of scheduling a set of jobs with different release dates, processing times and deadlines on a single machine without pre-emption. The optimization variants of this problem are ${\mathcal {N}}{\mathcal {P}}$-hard, be them minimizing maximum lateness or the number of tardy jobs with release dates. Existing exact approaches have limited scalability. They require large computational times and rely fully or partially on off-the-shelf solvers of mixed integer programs.

In real life industrial settings, access to these solvers could be prohibitively expensive and would require expert knowledge to implement. In addition, the characteristics of the jobs are assumed known and deterministic while they are generally good estimates based on historical data. Moreover, responsiveness is key while exact methods could be computationally expensive. Because of the aforementioned reasons, manufacturers are more inclined to implement heuristic approaches rather than exact methods. Heuristics explain and provide insight to the construction process of a schedule.

This paper proposes a simple but efficient enhancement of the search heuristic F. It augments F with an incremental search and a restart strategy. The proposed enhancements speed up F significantly, as evidenced by the experimental investigation. $\hbox {F}_{IR}$ outperforms existing (meta) heuristics and the constraint satisfaction state of the art IBM CP solver. Most importantly, $\hbox {F}_{IR}$ tackles the largest problem instances to date.

Future research related to $\hbox {F}_{IR}$ is multifold. First, the search of F can be accelerated by augmenting F with learning mechanisms that reflect the knowledge acquired during the exploitation of regions of the state-space. Second, $\hbox {F}_{IR}$ can be generalized to other real-life large-scale combinatorial problems such as scheduling in multiprocessor systems and scheduling precedence-related jobs. Third, $\hbox {F}_{IR}$ can be combined with other existing approaches for better performance. Fourth, it is worth investigating the theoretical convergence of $\hbox {F}_{IR}$ to existing feasible solutions could be useful. Last, investigating the impact of the stochastic versus deterministic behaviour of F on the performance of $\hbox {F}_{IR}$.
